# Chemotherapy-induced PTEN-L secretion promotes the selection of PTEN-deficient tumor cells

**DOI:** 10.1186/s13046-024-03059-y

**Published:** 2024-05-11

**Authors:** Ming Wang, Zhenzhen Pan, Xu Chu, Xiaohan Yao, Xixi Duan, Jiajia Wan, Xiaohan Lou, Wenqing Li, Yan Yan, Lin Chen, Junfeng An, Zhihai Qin

**Affiliations:** 1 Medical Research Center, The First Affiliated Hospital of Zhengzhou University, Zhengzhou University, Zhengzhou, Henan 450052 China; 2 The first affiliated hospital of Henan University of science and technology, Luo Yang, China; 3 Guangzhou DaAn Clinical Laboratory Center Co. Ltd, YunKang Group, Guangzhou, 510000 China

## Abstract

**Background:**

PTEN loss has been identified in various tumor types and is linked to unfavorable clinical outcomes. In addition to PTEN mutation, multiple mechanisms contribute to PTEN loss during tumor development. However, the natural selection process of PTEN-deficient tumor cells remains unclear. Here, we aimed at further elucidating the role of PTEN-L in tumor progression.

**Methods:**

PTEN knockout cell lines were generated using CRISPR/Cas9 technology. Ni-NTA affinity column chromatography was employed for PTEN-L purification. Tumor cell metastasis was evaluated in murine models and observed using the IVIS Spectrum Imaging System. RNA-sequencing, western blotting, PCR, flow cytometry, and cell proliferation assays were employed to investigate tumor cell dormancy and related mechanisms.

**Results:**

The chemotherapeutic drugs, cisplatin, paclitaxel, and doxorubicin, induced tumor cells to secrete PTEN-long (PTEN-L), which shields PTEN-deficient tumor cells from chemotherapy-induced apoptosis better than it shields PTEN-intact cells. Further investigation revealed that PTEN-L treatment induced dormancy in PTEN-null tumor cells, characterized by an increase in p16 and p27 levels, cell-cycle arrest, reduced cell proliferation, and enhanced DNA repair. Furthermore, PTEN-L treatment selectively promoted the accumulation and growth of PTEN-null tumor cells in the lungs of C57BL/6J mice, while evading immune surveillance. Mechanistically, PTEN-L induced dormancy in PTEN-null tumor cells by activating the p38 signaling pathway. Addition of a p38 inhibitor effectively reversed dormancy and growth of PTEN-deficient tumor cells in the lungs. We also demonstrated that PTEN expression played a pivotal role in determining the outcome of PTEN-L-mediated antitumor therapy.

**Conclusions:**

In summary, PTEN-L was identified as a potent inducer of dormancy in PTEN-deficient tumor cells, which increased their efficient selection within the tumor microenvironment.

**Supplementary Information:**

The online version contains supplementary material available at 10.1186/s13046-024-03059-y.

## Background

Genetic mutations play a pivotal role in malignant transformation and tumor progression. PTEN is among the most frequently mutated tumor suppressor genes, with documented instances of inactivation or deletion in a wide spectrum of tumors [1]. Intriguingly, deletion of a single copy of PTEN can lead to markedly reduced PTEN expression within tumor tissues. For instance, PTEN mutations have been reported in less than 5% of all breast cancer cases, while in 40% of these cases, PTEN expression is diminished or absent [2]. Extensive research has shown that non-genetic mechanisms, including epigenetic regulation, non-coding RNAs, phosphorylation, ubiquitination, and other post-translational modifications, contribute to PTEN loss [3]. However, how PTEN-deficient tumor cells survive and their natural selection process during tumor promotion and after therapy remain unclear.

PTEN-long (PTEN-L) is a translational variant of PTEN that contains an additional 173 amino acids at its N-terminus [4]. It is secreted from cells and can enter neighboring cells, facilitated by its distinctive sequence featuring six arginine residues. Notably, PTEN-L induces breast cancer cell death by counteracting the PI3K pathways [4]. Furthermore, PTEN-L treatment reduces the proliferation of HepG2 liver cancer cells while promoting apoptosis in these cells [5]. Despite the promising findings of studies investigating PTEN-L, its comprehensive antitumor effects have not been exhaustively elucidated in preclinical studies. Consequently, questions about PTEN-L function in various tumor types and its interaction with PTEN expression or PTEN-loss selection during tumor progression remain unresolved. Therefore, delving into the impact of PTEN-L on specific tumor types and unraveling the underlying mechanisms represent crucial steps in advancing preclinical studies of PTEN-L.

Tumor cell metastasis is the primary contributor to patient mortality. As the tumor cells travel from the primary tumor to distant organs, they must adapt their characteristics to endure immune attacks [6]. Zhang et al. revealed that primary tumor cells may lose PTEN expression upon dissemination into the brain, which is reversible upon exiting the brain microenvironment. Furthermore, reduced PTEN expression was linked to an unfavorable prognosis and a shorter time to brain recurrence [7]. These findings imply that PTEN expression plays a pivotal role in the fate of tumor cells during metastasis. Nevertheless, the role of PTEN-L in progression is yet to be explored, particularly in cases where PTEN-null cells were selected or PTEN expression was hindered in the metastatic microenvironment during the transfer from primary organs to metastatic sites.

The current study was aimed at elucidating the role of PTEN-L in tumor progression as well as the process by which PTEN-deficient tumor cells are naturally selected during tumor promotion.

## Methods

### Cell culture

The mouse mammary carcinoma cell line (EO771), mouse pancreatic carcinoma cell line (Pan02), and mouse immortalized embryonic fibroblasts (iMEFs) were maintained in our laboratory, while the mouse Lewis lung carcinoma cell line (LLC) was generously provided by Professor Yan Li’s group at the Academy of Military Medical Sciences in Beijing. The human malignant glioblastoma cell line U87MG was procured from ATCC (HTB-14). These cell lines were cultured in DMEM (Gibco, Carlsbad, CA, USA) supplemented with 10% FBS (Invigentech, Irvine, CA, USA), 100 IU/mL penicillin, and 100 mg/mL streptomycin (HyClone, Logan, UT, USA) at 37 °C with 5% CO2. Bone marrow-derived cells (BMDMs) were isolated from the tibia and femur of C57BL/6J mice and cultured in RPMI-1640 (Gibco) complete medium. The immortalized bone marrow-derived macrophages (iBMDMs) were cultured in RPMI-1640 complete medium.

### Animals

The C57BL/6J mice (female, 10 weeks old, weighing 20–22 g) and severe combined immunodeficiency (SCID) mice (female, 12 weeks old, weighing 20–22 g) utilized in this research were procured from Charles River (Beijing, China) and accommodated at the Experimental Animal Center of Zhengzhou University. The mice were kept under a temperature range of 20–25 °C with a 12-hour light-dark cycle.

### PTEN-L purification

PTEN-L purification followed established protocols, as previously published [8]. Recombinant plasmids with the PTEN-L sequence were introduced into Escherichia coli BL21, and the V451 strain, containing these plasmids, was induced for protein expression in 300 ml LB medium at 37 °C. Upon reaching an OD600 of approximately 0.6, 5 µM IPTG (I8070, Solarbio, Beijing, China) was added, and cultivation continued at 37 °C for 6 to 8 h. The harvested culture underwent purification using the His-tag Protein Purification Kit (P2226, Beyotime, Shanghai, China), with verification of purity through western blotting and Coomassie brilliant blue staining. Subsequently, the purified protein underwent procedures such as dialysis, concentration, and activity verification for use in subsequent experiments.

### Cell proliferation assay

Tumor cells were plated at a density of 2000 cells per well in 96-well plates, followed by the addition of PTEN-L (1 µg/ml) once cells adhered. At 24 h, 48 h, and 72 h, 10% CCK8 (CK-04, Dojindo, Kumamoto, Japan) was introduced, and cells were incubated for 2 h at 37 °C. Absorbance values at 450 nm were subsequently quantified using a microplate reader (ThermoFisher Scientific, Waltham, MA, USA).

### Cloning formation experiment

In the clone formation assay, 400 cells were initially seeded per well in a 6-well plate. The experimental group received 1 µg/ml of PTEN-L, while phosphate-buffered saline (PBS) served as the control. After ten days, the culture medium was discarded, and cells were washed with PBS. Subsequently, cells were fixed with 4% paraformaldehyde (PFA, Solarbio, Beijing, China) for 15 min at room temperature, followed by staining with 0.1% crystal violet (G1014, Servicebio, Wuhan, China) for 10 min. Excess dye was removed by washing with 1×PBS. Cell colony images were captured using the ChemiDoc MP imaging system from Bio-Rad, Hercules, CA, USA, and colony quantification was performed using Image J software.

### Apoptosis

To examine the impact of PTEN-L on tumor cells during chemotherapeutic drug treatment, cells were exposed to either conditional medium, PTEN-L (1 µg/ml), paclitaxel (PTX) at 100 nM (D8810, Solarbio), or the PTEN inhibitor SF1670 (HY-15,842, MCE, NJ, USA) at 300 nM. After 24 h, the Apoptosis Detection Kit (556,547, BD, Franklin Lakes, NJ, USA) was utilized for staining apoptotic and dead cells, and Annexin V^+^PI^+^ cells were analyzed using flow cytometry (FACSCanto II, BD).

### PTEN Knockout

PTEN knockout cell lines, including EO771-PTEN-KO, Pan02-PTEN-KO, LLC-PTEN-KO, iMEF-PTEN-KO, and iBMDM-PTEN-KO, were generated using CRISPR/Cas9 technology. The specific sgRNA sequences targeting PTEN were Pten-sgRNA-1-Forward: 5′- CACCGAGTCACAATTCCCAGTCAG-3′ and Pten-sgRNA-1-Reverse: 5′- AAACCTGACTGGGAATTGTGACTC-3′. The sgRNA cloning vector utilized was lentiCRISPR v2-AmCyan. Cells were transfected with plasmids containing the sgRNA sequences using Lipofectamine 3000 transfection reagent (L3000015, Invitrogen, Waltham, MA, USA). Single clones were screened, amplified, and subsequently employed in downstream experiments.

### Tumor cell labeling

The EO771-PTEN-KO-GFP-Luc cell line was established through lentiviral transduction following the manufacturer’s protocol. Additionally, EO771-mCherry cells expressing red fluorescent protein (mCherry) were generated using the identical method. Lentivirus vectors were procured from Genechem (Shanghai, China), and the cell populations expressing the desired fluorescent proteins were subsequently isolated via cell sorting.

### Flow cytometry

To assess the expression of p16 and p21 in tumor cells within lung tissue, single-cell suspensions were prepared by digesting lung tissue with collagenase D (LS004188, Worthington, Lakewood, New Jersey, USA). For intracellular staining of p16 and p21, cells were fixed and permeabilized using fixation/permeabilization buffer (00-5523-00, Invitrogen) for 10 min, followed by washing with 1× permeabilization washing buffer. The collected cells were blocked with 1% BSA in PBS for 30 min. Subsequently, primary antibodies p16 (1:100, 10883-1-AP, Proteintech, Wuhan, China) and p21 (1:100, sc-6246, Santa Cruz Biotechnology, Santa Cruz, CA, USA) were used to stain the cell suspensions at 4 °C for 30 min. Alexa Fluor 647-labeled rabbit anti-mouse IgG (H + L) (1:200, A21236, Invitrogen) and Alexa Fluor 647-labeled mouse anti-rabbit IgG (H + L) (1:200, A31573, Invitrogen) served as secondary antibodies for subsequent staining. Following all staining steps, cells were washed twice with 1× PBS. Data analysis was conducted using a BD FACSCanto II flow cytometer and FlowJo v10 software.

For the assessment of PTEN-L impact on immune cells in lung tissue, the tissue was digested and converted into a single-cell suspension. Specific primary antibodies were employed to stain distinct cell populations, including CD206^+^ macrophages (CD45^+^F4/80^+^CD206^+^), MHCII-expressing cells (CD45^+^F4/80^+^MHCII^+^), myeloid-derived suppressor cells (MDSCs) (CD45 + CD11b^+^Gr-1^+^), CD4 T cells (CD45^+^CD4^+^), CD8 T cells (CD45^+^CD8^+^), B cells (CD45^+^B220^+^), and NK cells (CD45^+^NK1.1^+^). Antibodies used are listed in supplementary Table 1.

### Western blot

For cellular or tissue total protein extraction, cells were lysed in RIPA lysis buffer (P0013C, Beyotime, Shanghai, China) supplemented with phosphatase and protease inhibitors cocktail (P1049, Beyotime). Cell membrane proteins and cytoplasmic proteins were extracted using cell membrane protein and cytoplasmic protein extraction kits (P0033, Beyotime), while nuclear proteins were extracted using a nuclear protein extraction kit (P0028, Beyotime). Protein concentration was determined using the BCA protein assay kit (23,225, ThermoFisher Scientific) following the manufacturer’s instructions. Subsequently, 20 µg of protein samples were electrophoresed on a 10% SDS-PAGE gel (PG112, EpiZyme Biotechnology, Shanghai, China), transferred to a Hybridization Nitrocellulose filter membrane (HATF00010, Millipore, Billerica, MA, USA), and blocked at room temperature for 1 h in 1×TBS-T (TBS with 0.01% Tween-20) containing 5% skim milk. The membrane was then incubated with primary antibodies (supplementary Table 2) overnight on a shaker at 4 °C. After washing, the membrane was incubated with secondary antibody (AS014 or AS003, ABclonal, 1:3000) at room temperature for 1 h. Enhanced Chemiluminescent reagent (P10300, NCM Biotech) was applied to the membrane, and bands were visualized using the ChemiDoc MP imaging system (Bio-Rad, Hercules, CA, USA).

### PCR

Total RNA was isolated from cells using Trizol reagent (9109, Takara, Tokyo, Japan), followed by reverse transcription into cDNA using the PrimeScript RT Master Mix (RR036A; Takara) in accordance with the manufacturer’s instructions. Real-time quantitative polymerase chain reaction (PCR) was conducted using the TB Green Premix Ex Taq II reagent (RR820A, Takara), and detection was performed using the Agilent Mx3005P instrument. RNA expression levels were determined using the 2^(-ΔΔCt) method, with β-actin serving as the reference gene for normalization. Primer sequences for mRNAs are provided in supplementary Table 3.

### Animal experiments

To investigate the impact of PTEN-L on tumor cell metastasis in the lungs, 5 × 10^5^ EO771-mock or EO771-PTEN-KO cells were injected into C57BL/6J mice, and 5 × 10^5^ EO771-PTEN-KO cells were injected into SCID mice via the tail vein. On the third day, mice were intraperitoneally administered 4 mg/kg of PTEN-L, with PBS as a control. Lung tissues were collected for hematoxylin and eosin (HE) staining. Furthermore, to assess the influence of PTEN-L on PTEN-wide type or PTEN loss tumor cell metastasis, a total of 1 × 10^6^ mCherry-labeled EO771 cells and GFP-Luc-labeled EO771-PTEN-KO cells (mixed at a 1:1 ratio) were injected into C57BL/6J mice via the tail vein. Tumor cell numbers in the lungs were detected by flow cytometry on days 1, 3, and 7, the mCherry and luciferin signals were observed after D-luciferin injection (150 mg/kg body weight). Fluorescence quantification was conducted using the IVIS Spectrum Imaging System (Caliper Life Sciences, Hopkinton, MA, USA) at day 21, and fluorescence intensity within regions of interest was analyzed using Living Image 4.4 software.

To study the impact of PTEN-L on lung macrophages, C57BL/6J mice were intraperitoneally injected with PTEN-L (4 mg/kg), with PBS as the negative control. After 48 h, macrophages from lung, spleen, liver, and brain tissues were analyzed using flow cytometry. To explore the effect of chemotherapy drugs on PTEN-L secretion, C57BL/6J mice were treated with DDP (5 mg/kg), PTX (10 mg/kg), or DOX (5 mg/kg). After 48 h, mice were euthanized, and PTEN-L levels in the liver, spleen, and lung were analyzed using western blot experiments.

To investigate the impact of p38 inhibition on tumor cell lung colonization, 5 × 10^5^ EO771-PTEN-KO tumor cells were injected via the tail vein and treated with either 4 mg/kg PTEN-L, SB202190 (MCE, 5 mg/kg), or a combination. C57BL/6J mice were euthanized at day 21, and lung tissues were collected for HE staining. To determine if PTEN-L treatment could enhance the anti-tumor effect of chemotherapy, we established a subcutaneous tumor model by injecting 1 × 10^6^ EO771-PTEN-KO cells in C57BL/6J mice. Once the tumor size reached 100 mm^3^ on day 13, the mice were randomly divided into four groups (*n* = 8 per group). The mice then received treatments of PTEN-L (4 mg/kg), PTX (10 mg/kg), or a combination thereof. Tumor sizes were determined using the formula: 1/2× length (L) × width (W)^2^. On day 25, lung tissues were collected for hematoxylin and eosin (HE) staining to assess metastasis.

### RNA-Seq

To analyze the distinct mRNA expression profiles of EO771-PTEN-KO cells treated with PBS and PTEN-L (1 µg/ml, 3 h), RNA-sequencing was conducted using Illumina HiSeq 2500 (OE Biotech, Shanghai, China). DESeq2 software normalized gene counts in each sample, and differential protein-coding genes were selected based on fold change and significance tests. Differential gene expression with statistical significance was defined as *p* < 0.05.

### Statistics

Statistical analysis utilized GraphPad Prism 9 software, and data were expressed as mean ± standard deviation (SD). Unpaired t-test was employed for two-group comparisons, while one-way or two-way ANOVA was utilized for multiple comparisons. Statistical significance was indicated by asterisks (*), where *p < 0.05, **p < 0.01, and ***p < 0.001.

## Results

### Chemotherapy-induced PTEN-L secretion protects PTEN-null tumor cells from apoptosis

To investigate the distinct effects of PTEN-wild type or PTEN-null tumor cells, we initially established PTEN-knockout (KO) cell lines using the CRISPR-Cas-9 system in the tumor cell lines LLC, EO771, Pan02, iBMDMs, and iMEFs (Supplementary Fig. 1A). PTEN-L was purified using a 6-His plasmid system. The purity of PTEN-L was confirmed using Coomassie brilliant blue staining and western blot (Supplementary Fig. 1B). The cell permeability of PTEN-L was verified by detecting PTEN-L expression in the cell membrane, plasma, and nuclei of U87MG glioblastoma cells lacking PTEN expression (Supplementary Fig. 1C). The phosphatase activity of PTEN-L was confirmed by its ability to reduce AKT phosphorylation (Supplementary Fig. 1D).

**Fig. 1:**
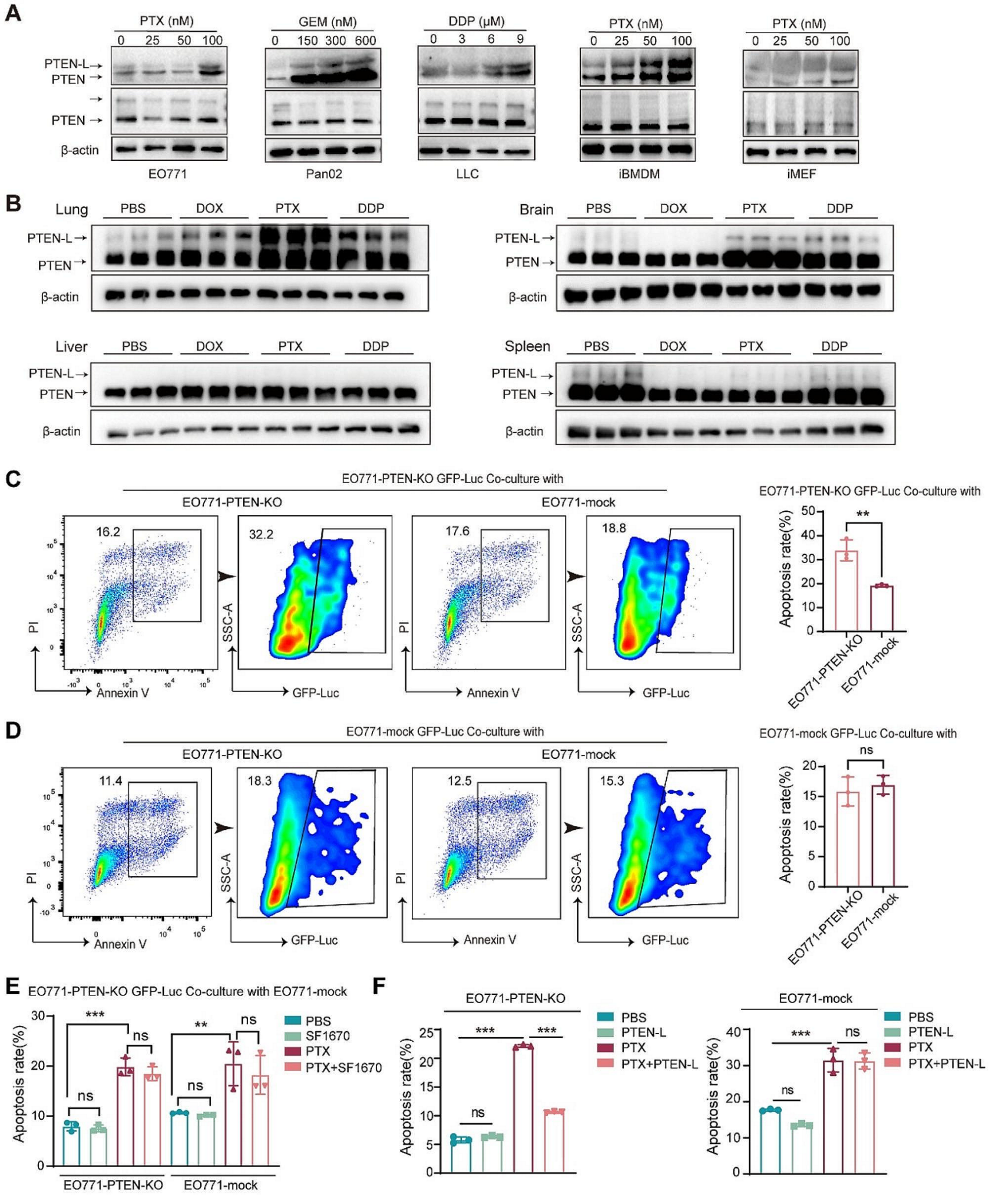
PTEN-L inhibits PTX-induced apoptosis in PTEN-null tumor cells. (**A**) EO771, Pan02, LLC, iBMDM, and MEF were subjected to 24-h treatments with PTX, GEM, and DDP at specified concentrations. Subsequently, cell conditioned media and extracts underwent immunoblotting with antibodies against PTEN, with representative images provided. β-actin served as the loading control for cell extracts. (**B**) C57BL/6J mice received treatments of DOX (5 mg/kg), PTX (10 mg/kg), or DDP (5 mg/kg) over a 48-h period. Western blot analysis was employed to detect PTEN/PTEN-L in tissue lysates from the lung, liver, brain, and spleen, with representative images presented. (*n* = 3) (**C, D**) GFP-Luc labeled EO771-PTEN-KO cells (upper panel) and EO771-mock GFP-Luc cells (lower panel) were co-cultured with unlabeled EO771-mock or EO771-PTEN-KO cells at a 1:1 ratio. Post 24-h PTX (100 nM) treatment, the proportion of apoptotic GFP-Luc labeled cells was determined via flow cytometry. The gating strategy is depicted on the left, and the results are summarized in bar plots from three independent experiments (mean ± SD, Welch’s t-test). (**E**) EO771-PTEN-KO GFP-Luc cells were co-cultured with unlabeled EO771-mock cells at a 1:1 ratio and subsequently treated with PTX (100 nM) alone or in combination with the PTEN inhibitor SF1670 (300 nM) for 24 h. Apoptosis in both cell populations was assessed, and the results from three independent experiments are summarized in bar plots (Welch’s t-test). (**F**) EO771-PTEN-KO (left panel) and EO771-mock (right panel) cells were treated with PBS, PTEN-L (1 µg/ml), PTX (100 nM), or a combination thereof, for 24 h. The percentage of apoptotic cells was calculated (*n* = 3); one-way ANOVA; ns: not significant

When investigating the mechanism underlying PTEN-L secretion, we first found that cisplatin (DDP), paclitaxel (PTX), and doxorubicin (DOX) upregulated PTEN-L secretion in cell-conditioned media (CM) without reducing the basal level of PTEN/PTEN-L in the cell lysates (Fig. 1A). Consistently, DDP, PTX, and DOX also significantly upregulated PTEN-L expression in the tissue lysates of lungs, but not in the brain, liver, or spleen (Fig. 1B).

Next, we demonstrated that the supernatants of EO771 cells, iMEFs, and iBMDMs, which contained high amounts of PTEN-L after PTX stimulation, could protect EO771-PTEN-KO cells from apoptosis after DDP treatment (Supplementary Fig. 2A-D). Similarly, EO771-mock cells could protect EO771-PTEN-KO cells from PTX induced apoptosis, which was nullified when the PTEN inhibitor, SF1670, was added (Fig. 1C-E). Moreover, the purified PTEN-L had a similar protective effect against PTX-induced apoptosis in EO771-PTEN-KO tumor cells (Fig. 1F). These results further confirmed that exogenous PTEN-L inhibited apoptosis in PTEN-null tumor cells during chemotherapy.

### PTEN-L induces cell-cycle arrest in PTEN-null tumor cells

**Fig. 2:**
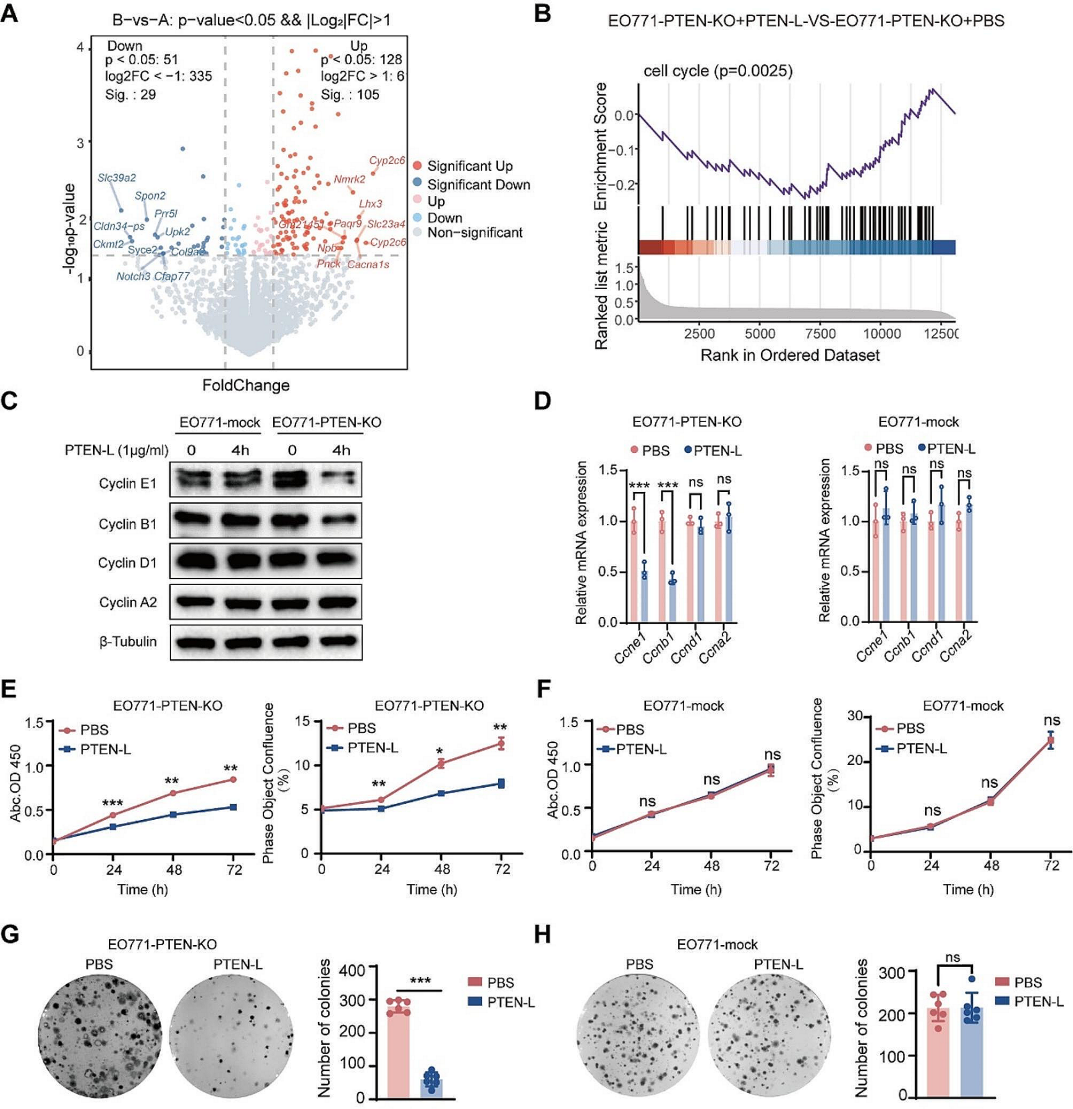
PTEN-L induces cell cycle arrest in PTEN-null tumor cells. **A-B**: EO771-PTEN-KO cells were administered PTEN-L (1 µg/ml) for 3 h. The RNA-seq volcano plot illustrates the differentially expressed genes, with red and blue dots signifying significantly upregulated or downregulated genes, respectively (*p* < 0.05). (**B**) GSEA revealed a pronounced enrichment of cell cycle-related genes. (**C-D**) EO771-PTEN-KO and EO771-Mock cells were exposed to PTEN-L (1 µg/ml) for 4 h. The expression levels of cyclins, both at the protein and mRNA stages, were quantified via western blot (left) and RT-PCR (right) (*n* = 3); Welch’s t-test. (**E-H**) Treatment of EO771-PTEN-KO and EO771-Mock cells with PTEN-L (1 µg/ml) led to analyses of cell proliferation using either the CCK8 assay (measuring OD450) or Incucyte (assessing cell confluence). (**G-H**) The cell fusion assay was employed to evaluate the cells’ colony formation potentials. Representative images are displayed on the left, and bar plots summarize the results from six independent experiments. Welch’s t-test; *p < 0.05, **p < 0.01; ***p < 0.001; ns: not significant

To demonstrate that PTEN-L protects PTEN-deficient tumor cells from apoptosis, we investigated the underlying mechanisms. Through RNA sequencing (RNA-seq) analysis, we identified 134 significantly altered genes in PTEN-L-treated EO771-PTEN-KO cells (Fig. 2A). Tumor cells can counteract chemotherapy by evading cell-cycle arrest. Gene set enrichment analysis revealed significant enrichment of cell cycle-related genes (Fig. 2B). Moreover, PTEN-L treatment markedly induced cell-cycle arrest by downregulating key enzymes in the cell cycle, particularly cyclins E1 and B1 (Fig. 2C-D, Supplementary Fig. 3A-B, G-H). Cell-cycle arrest was further validated by the reduced rate of tumor cell proliferation observed in all three PTEN-KO cell lines (Fig. 2E, Supplementary Fig. 3D, J). Additionally, the diminished colony formation rate suggested the decreased proliferative capacity of PTEN-null tumor cells upon PTEN-L treatment (Fig. 2G, Supplementary Fig. 3F, L). Importantly, PTEN-L treatment had no impact on the proliferation or colony formation of PTEN-mock tumor cells (Fig. 2F, H, Supplementary Fig. 3C, E, I, K).

### PTEN-L selectively induces the colonization and growth of PTEN-null tumor cells in the lungs

**Fig. 3:**
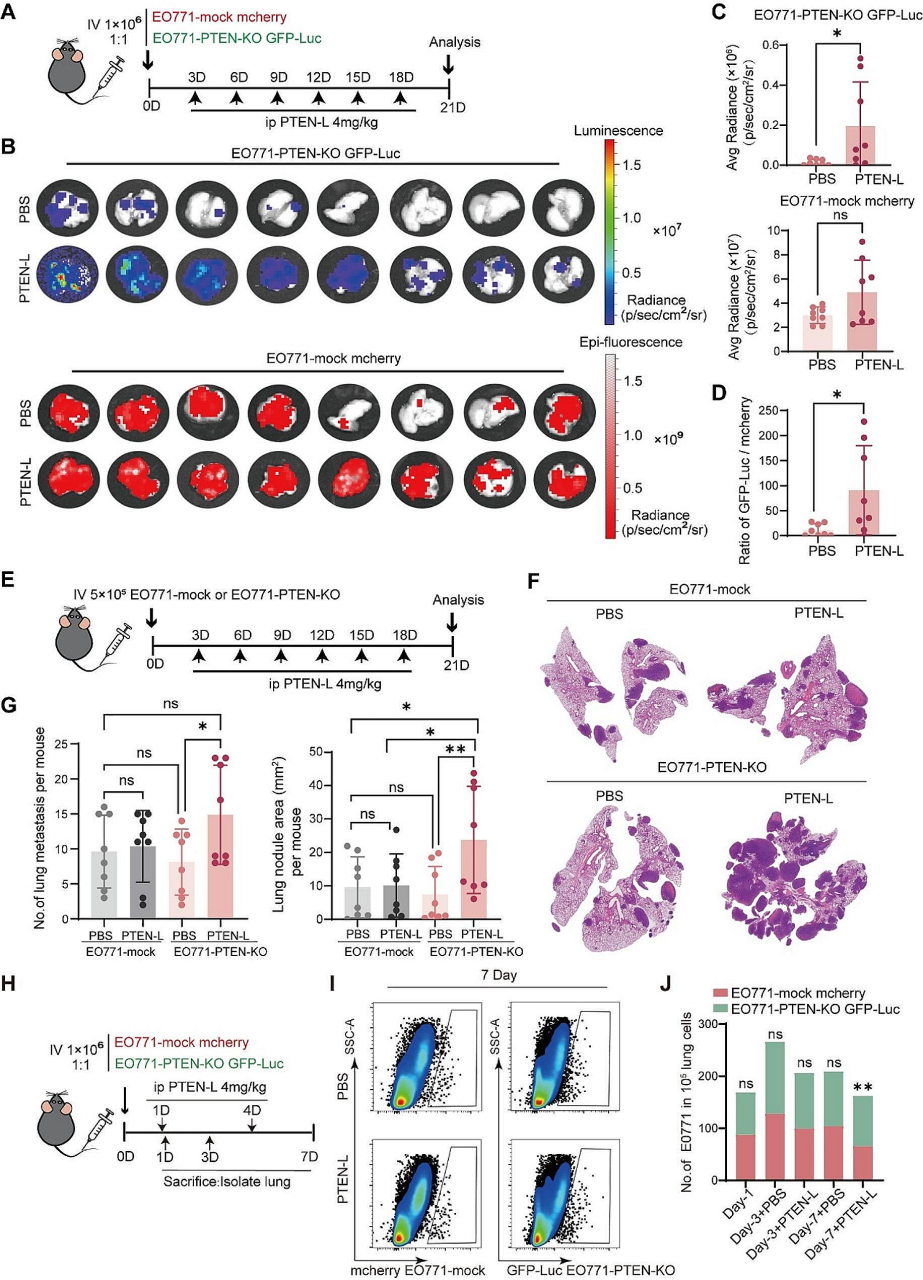
PTEN-L selectively induces PTEN-null tumor cells colonization and outgrowth in the lung. **A-D**: A total of 1*10^6^ EO771-mock mCherry and EO771-PTEN-KO GFP-luc cells (mixed at a 1:1 ratio) were intravenously injected into C57BL/6J mice. PTEN-L (4 mg/kg) was administered every 3 d. (**A**) Schematic representation of the experimental design. (**B**) IVIS imaging displays the signals of GFP-Luc and mCherry in the lungs. (**C**) Fluorescence intensity quantification of EO771-PTEN-KO GFP-Luc (upper) and EO771-mCherry (lower) cells in lung tissues (*n* = 8); Unpaired t-test. (**D**) Ratio of GFP-Luc to mCherry fluorescence signals. E-G: A total of 1*10^6^ EO771-mock or EO771-PTEN-KO cells were injected into the tail vein of C57BL/6J mice, with PTEN-L (4 mg/kg) treatment every 3 d. (**E**) Schematic of the experimental design. (**F**) HE staining illustrates representative lung tissues from each group. (**G**) Quantitative analysis of the number (left) and total area (right) of lung metastatic nodules across four groups of mice (*n* = 8); one-way ANOVA. *p < 0.05), **p < 0.01. **H-J**: A total of 1*10^6^ EO771-mock mCherry and EO771-PTEN-KO GFP-luc cells (mixed at a 1:1 ratio) were injected into the tail vein of C57BL/6J mice. PTEN-L (4 mg/kg) was administered on days 1 and 4, and mice were sacrificed on days 1, 3, and 7. Lung single-cell suspensions were analyzed via flow cytometry. (**H**) Schematic of the experimental design. (**I**) Images depicting the labeled EO771 tumor cells in each group. (**J**) Calculation of the relative ratio of EO771-PTEN-KO GFP-Luc to EO771 mCherry cells (*n* = 5), two-way ANOVA, **p < 0.01

We determined whether PTEN-L-induced cell-cycle arrest could lead to the selection of PTEN-null tumor cells in vivo. We established an in vivo tracking system by injecting a mixture of mCherry-labeled EO771-mock and GFP-Luc-labeled EO771-PTEN-KO cells into the tail vein (Fig. 3A). PTEN-L treatment resulted in markedly increased GFP-Luc signals, whereas mCherry signals were largely unaffected (Fig. 3B, C). The ratio of GFP-Luc/mCherry fluorescence was significantly elevated in the PTEN-L-treated lungs (Fig. 3D).

To validate these findings and eliminate the potential confounding effects of fluorescent proteins on tumor cells, we established an in vivo lung metastasis model by injecting EO771-mock or EO771-PTEN-KO cells (Fig. 3E). Conversely, PTEN-L treatment significantly increased the number and size of lung nodules in EO771-PTEN-KO cells but not in the PTEN-mock cells (Fig. 3F, G). We also investigated the number of injected tumor cells in the lungs (Fig. 3H). On days 1 and 3, there was no significant difference in the numbers of EO771-mock and EO771-PTEN-KO cells. However, the EO771-PTEN-KO/EO771-mock ratio increased significantly after PTEN-L treatment on day 7 (Fig. 3I, J). This suggests that PTEN-L enhances the survival of PTEN-null tumor cells in the lungs during the early stages of tumor cell colonization.

### PTEN-L-induced immune escape plays a major role in the selection of PTEN-null tumor cells

**Fig. 4:**
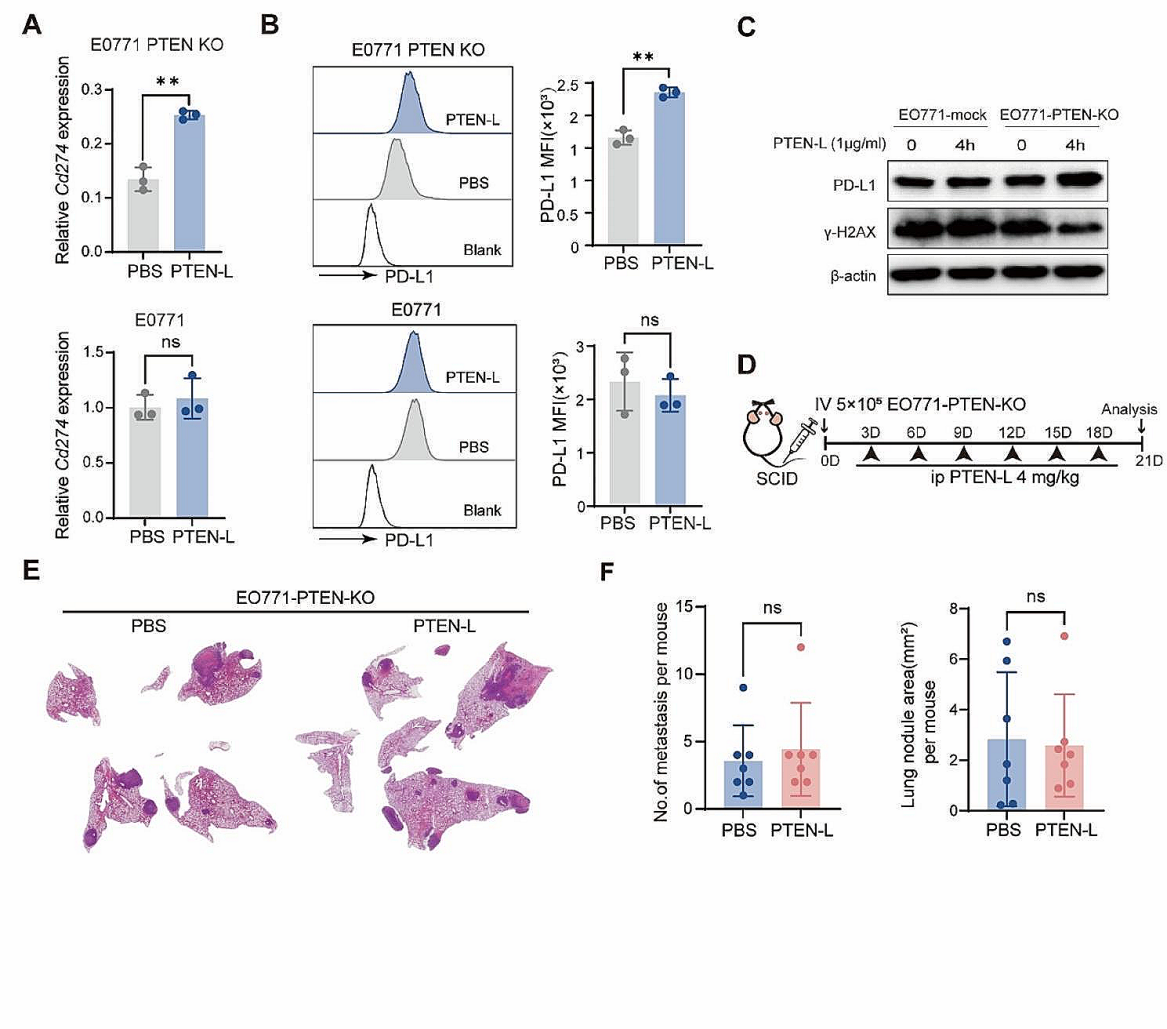
PTEN-L induces PTEN-null tumor cells immune escape. **A-C**: EO771-PTEN-KO (upper) and EO771-mock (lower) cells underwent PTEN-L treatment (1 µg/ml) for varying durations. (**A**) The experimental design is depicted schematically. (**B**) RT-PCR was employed to assess the mRNA expression of *Cd274*. (**C**) The MFI of PD-L1 was quantified by flow cytometry 24 h post-treatment (*n* = 3), Welch’s t-test. (**D**) Western blot analysis revealed the expression levels of PD-L1 and γ-H2AX following a 4-h treatment. **D-F**: A total of 5* 10^5^ EO771-PTEN-KO cells were intravenously administered into the tail vein of SCID mice, followed by PTEN-L treatment (4 mg/kg) every 3 d. (**D**) The experimental setup is outlined. (**E**) HE staining showcases representative lung histopathology from two groups. (**F**) The number and area of lung metastatic lesions were analyzed statistically (*n* = 7), Welch’s t-test

Immune attack is a key mechanism for eliminating tumor cells [9]. Further experiments confirmed that PTEN-L treatment significantly upregulated the expression of the key immune escape molecule PD-L1 in EO771-PTEN-KO cells (Fig. 4A-C). In vivo experiments further confirmed that PTEN-L treatment enhances the expression of PD-L1 in EO771-PTEN-KO tumor cells within lung metastatic nodules following the treatment. (Supplementary Fig. 4A). Furthermore, DNA repair plays a role in the immune surveillance of tumor cells by inhibiting the release of new antigens and immunogenic cell death [10, 11]. We demonstrated that PTEN-L could confer protection against DNA damage in EO771-PTEN-KO cells by downregulating γ-H2AX, which responds to double-stranded DNA breaks (Fig. 4C). This downregulation potentially prevents innate immune responses from targeting tumor cells. Moreover, excluding tumor cells, the PTEN-L treatment could also alter the phenotype of macrophages. PTEN-L treatment upregulated *Mrc1* and *Tgfb* expression and downregulated *Tnfa*, *Il6*, and *Inos* expression in BMDMs; (Supplementary Fig. 5A). PTEN-L treatment may also prompt BMDMs to adopt an immunosuppressive phenotype characterized by the upregulation of markers such as F4/80, CD11b, and CD206 (Supplementary Fig. 5B). In line with the findings, PTEN-L treatment was observed to significantly increase the population of F4/80^+^CD206^+^ M2 macrophages within the pulmonary environment (Supplementary Fig. 5C-F). To delve deeper into whether effective immunosurveillance can selectively promote the survival of PTEN-KO EO771 tumor cells in the lungs, the experiment was replicated using severe combined immunodeficiency (SCID) mice. However, PTEN-L treatment did not increase the number or size of EO771-PTEN-KO nodules in the lungs (Fig. 4D-F). These results further suggested that immune cells play a major role in inducing the growth of PTEN-null tumor cells in the lungs.

### PTEN-L induces selection of PTEN-null tumor cells via p38 signaling activation

**Fig. 5:**
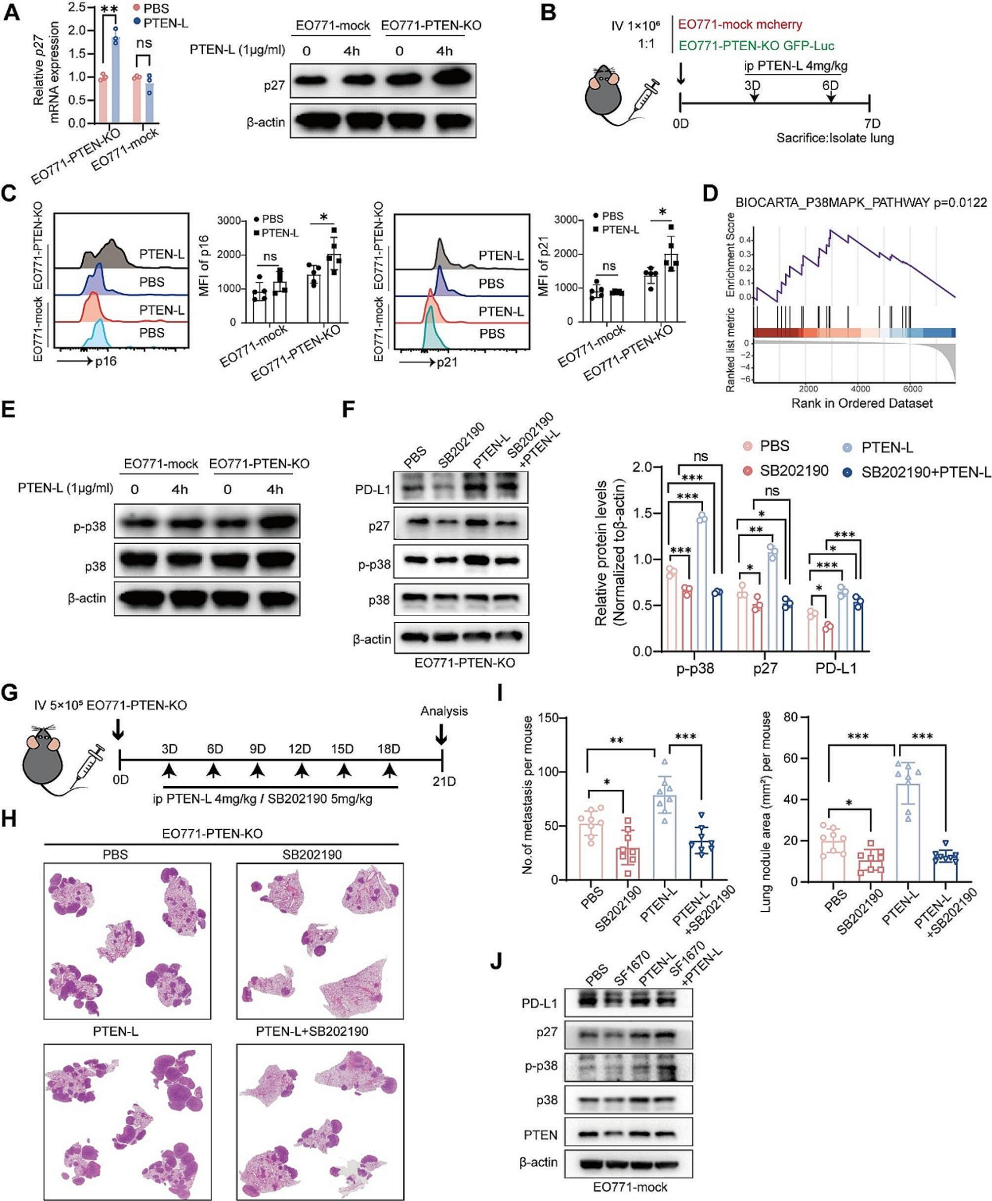
PTEN-L induces dormancy in PTEN-deficient tumor cells through the activation of p38. (**A**) EO771-PTEN-KO and EO771-mock cells were treated with PTEN-L (1 µg/ml) for various durations. The relative mRNA levels of *p27* were detected using RT-PCR (left), and protein levels were assessed by western blot (right). (**B**) A total of 1* 10^6^ EO771-mock mCherry and EO771-PTEN-KO GFP-luc cells (mixed at a 1:1 ratio) were injected into the tail vein of C57BL/6J mice. PTEN-L (4 mg/kg) was administered on days 3 and 6, and mice were sacrificed on day 7. The MFI of p16 and p21 was analyzed using flow cytometry. Repetitive histograms and bar plots are shown (*n* = 5), two-way ANOVA, *p < 0.05. (**C**) GSEA revealed the enrichment of p38-MAPK and ERK signaling pathways in genes related to PTEN-L-treated EO771-PTEN-KO cells. (**D**) The expression of phosphorylated p38 protein in EO771-PTEN-KO or EO771-mock cells post PTEN-L treatment was validated by western blot. (**E**) Western blot analysis was conducted to examine the expression of PD-L1, p-p38, and p27 proteins in EO771-PTEN-KO cells treated with PTEN-L (1 µg/ml) and/or SB202190 (10 nM) for 4 h. Quantification results from three experiments are shown below; one-way ANOVA, *P < 0.05; **P < 0.01; ***P < 0.001. F-H: A total of 5* 10^5^ EO771-PTEN-KO cells were injected into the tail vein of C57BL/6J mice. Treatments with PTEN-L (4 mg/kg) and/or SB202190 (5 mg/kg) were administered intraperitoneally every 3 d. (**F**) Schematic of the experimental design. (**G**) Representative HE staining results of lung metastases from the four treatment groups. (**H**) Statistical analysis of the number of lung metastatic foci (left) and the total metastatic area (right) ((*n* = 8), one-way ANOVA. (**I**) Expression analysis of PD-L1, p-p38, and p27 in EO771 cells treated with the PTEN inhibitor SF1670 (300 nM), PTEN-L (1 µg/ml), or a combination thereof. Representative western blot images are provided

PTEN-L induces cell-cycle arrest, inhibits proliferation, enhances DNA repair, and confers chemoresistance and immune escape, all of which are characteristics of cell dormancy [12]. We confirmed that PTEN-L treatment significantly activated the major dormancy-related protein p27 in PTEN-null tumor cells at both the mRNA and protein levels but had no clear effect on PTEN-wild-type cells (Fig. 5A, Supplementary Fig. 6A-B). Similarly, PTEN-L treatment upregulated p16 and p21 in EO771-PTEN-KO cells in the lungs (Fig. 5B-C). Further RNA-seq data revealed that the p38 signaling pathway, which regulates dormancy, was significantly enriched after PTEN-L treatment (Fig. 5D). This was confirmed by the increased phosphorylation of p38 (Fig. 5E, Supplementary Fig. 6C-D). Moreover, SB202190, a p38 inhibitor, significantly reversed the PTEN-L-induced upregulation of p27 and PD-L1(Fig. 5F), as well as the cell proliferation inhibition in EO771-PTEN-KO cells (Supplementary Fig. 6E). Additionally, the combination of PTEN-L and SB202190 significantly inhibited the growth of EO771-PTEN-null tumor cells in the lungs (Fig. 5G-I). These results confirmed that p38 inhibition suppressed PTEN-L-induced PTEN-null tumor cell proliferation in the lungs. Similarly, in PTEN inhibitor SF1670-treated EO771 cells, PTEN-L treatment significantly upregulated p38 activation and p27 expression (Fig. 5J). This further suggests that PTEN loss or inactivation is essential for the activation of p38 signaling and tumor cell dormancy during PTEN-L treatment.

### PTEN-L does not exhibit any antitumor effect on PTEN-null tumor cells in vivo

**Fig. 6:**
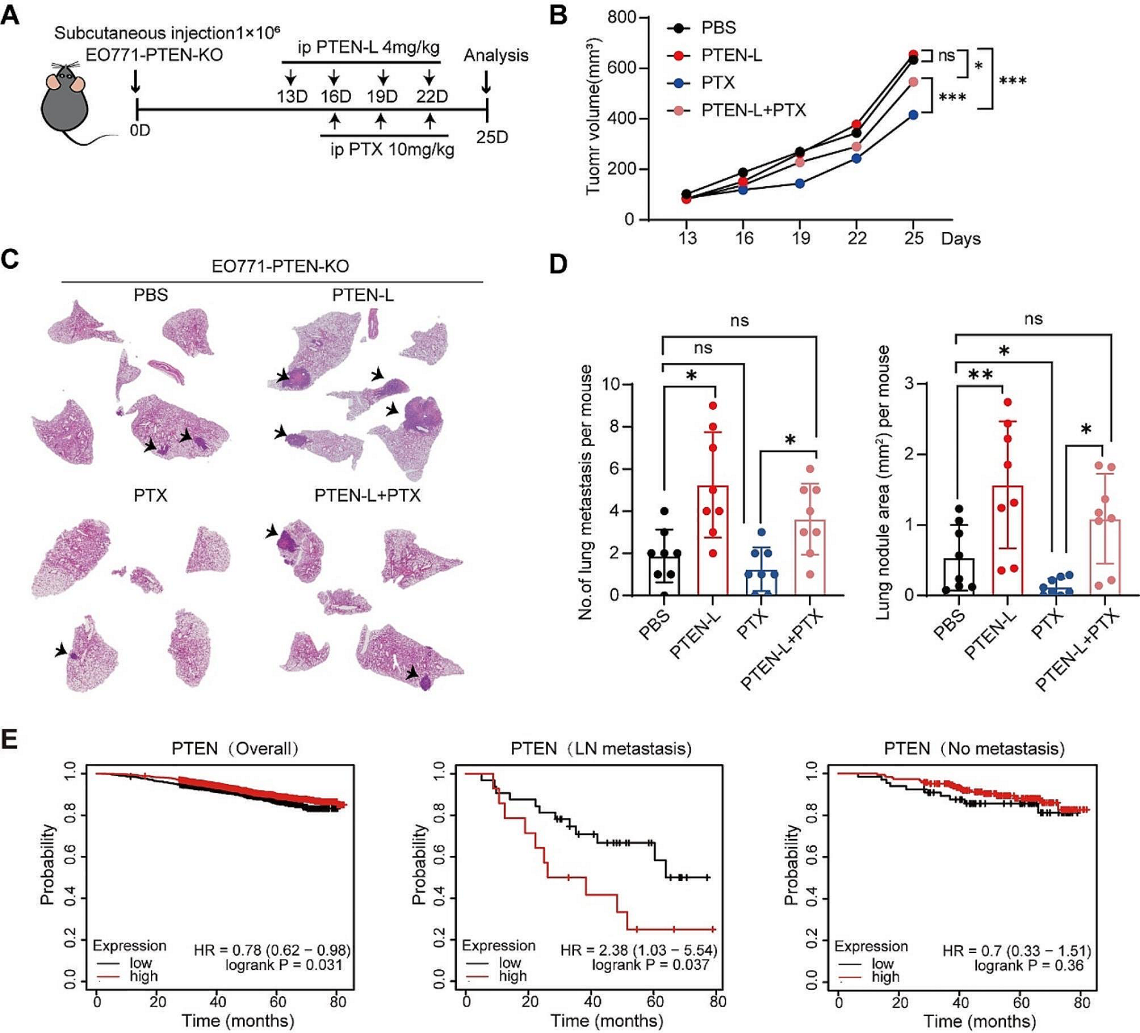
PTEN-L does not exhibit any anti-tumor effect on PTEN-null tumor cells in vivo. **A-D**: A total of 1*10^6^ EO771-PTEN-KO cells were subcutaneously injected into mice. Treatments with PTEN-L (4 mg/kg) or a combination with PTX (10 mg/kg) were administered intraperitoneally every 3 d. (**A**) Schematic diagram of the experimental design. (**B**) Tumor volume measurements are presented. (**C**) The HE staining images illustrate the histopathological changes in lung tissues. (**D**) Bar plots depict the number (left) and area (right) of lung metastases across the four groups (*n* = 8), mean ± SD; one-way ANOVA; *p < 0.05, **p < 0.01. (**E**) Overall survival analysis of breast cancer patients based on PTEN expression, as sourced from the TCGA database. The analysis is segmented into: General survival based on PTEN expression (Left); Survival of patients with lymph node metastasis (Middle); and Survival of patients without lymph node metastasis (Right)

Having demonstrated that PTEN-L selectively induces lung colonization and growth of PTEN-null tumor cells, we next compared the effects of PTEN-L and chemotherapeutic drugs on tumor promotion. Therefore, we established a subcutaneous tumor model using EO771-PTEN-KO tumor cells. C57BL/6J mice were treated with either PTEN-L or PTX (Fig. 6A). Surprisingly, in contrast to the previously reported antitumor effect of PTEN-L on 4T1 tumor cells [4], PTEN-L treatment did not inhibit the growth of EO771-PTEN-KO cells but led to a higher number of metastatic colonization in the lungs (Fig. 6B-D).

Finally, we analyzed the clinical relevance of PTEN expression in breast tumor metastasis. By examining The Cancer Genome Atlas dataset, we confirmed that PTEN expression was positively correlated with the overall survival of breast cancer patients, which is consistent with most existing literature [13]. However, in patients with lymph node metastasis, those with high PTEN expression had significantly shorter durations of survival, although this did not affect those without lymph node metastasis (Fig. 6E). These results further suggest that PTEN-L may negatively impact the outcomes of tumor therapy, especially in PTEN-deficient tumor cells.

## Discussion

Loss of PTEN has been documented across diverse neoplastic entities and is positively correlated with malignant tumor progression [14]. Despite the presence of PTEN mutations, various mechanisms are known to contribute to the loss or downregulation of PTEN in tumor cells [15]. Nonetheless, a comprehensive understanding of the survival and selection of PTEN-deficient cells during tumor treatment has not been achieved yet. In this study, we demonstrated that the secreted PTEN subtype PTEN-L exerts distinct effects on both PTEN-wild-type and PTEN-deficient tumor cells. PTEN-L treatment induced dormancy-related characteristics in PTEN-null tumor cells, including cell-cycle arrest, proliferation inhibition, chemoresistance, and immune escape. These characteristics enabled PTEN-null tumor cells to survive and grow in distinct microenvironments. Thus, this study accentuates a PTEN-L-dependent mechanism governing PTEN-loss selection during tumor metastasis, thereby highlighting the pivotal influence of PTEN expression in tumor cells on the efficacy of PTEN-L-based antitumor therapy.

The principle of natural selection is a cornerstone of cancer progression, where cells endowed with advantageous genetic and epigenetic characteristics gain a proliferative advantage. The concept is well-documented in the literature [16]. As tumors evolve, the immune system engages in a relentless battle against cancerous cells. Yet, some cells develop strategies to elude immune surveillance, enabling their survival and proliferation in distant organs [17]. Our findings demonstrate that PTEN-deficient tumor cells can endure and establish themselves in the lungs post-PTEN-L treatment. Notably, the metastatic potential of PTEN-mock and PTEN-KO EO771 cells exhibits no disparity in immunodeficient mice, implying that natural selection is not the sole driver of PTEN-KO tumor cell metastasis. In contrast, PTEN-L treatment or the secretion of PTEN-L induced by chemotherapy seems to promote the survival and pulmonary colonization of PTEN-KO tumor cells. Further investigations reveal that PTEN-L treatment elevates PD-L1 expression and curtails DNA damage, which may shield against immune cell-mediated eradication. Such insights lead us to postulate that PTEN-L modulates the selective survival of PTEN-KO tumor cells.

PTEN mutations are contribute to tumor malignancy. Numerous studies have reported that PTEN is a tumor suppressor gene that inhibits tumor cell proliferation and progression. Patients with breast cancer and high PTEN expression showed significantly prolonged survival [18]. In alignment with the prevailing body of literature, our Kaplan–Meier plot analysis reveals that patients with low PTEN expression levels have a markedly reduced overall survival rate when compared to those with high PTEN expression levels, especially in cases without lymph node metastasis. Conversely, among patients with lymph node metastasis, a higher PTEN expression level correlates with decreased survival times. This suggests that elevated PTEN expression level does not necessarily bestow a survival benefit in the context of lymph node metastasis; instead, it may, in fact, aggravate tumor metastasis in certain cases.

Loss of PTEN has been reported in multiple tumor types. PTEN exhibits a high mutation rate in prostate cancers [19]. Notably, the frequency of PTEN deletion is 10–20% in primary prostate cancers but increases to 30–45% in metastatic tumors [20]. PTEN loss is considered an early prognostic marker of prostate cancer metastasis [19]. Moreover, it is associated with advanced tumor stages and resistance to therapy. However, a tumor-promoting function of PTEN has also been reported. Fortin et al. reported that PTEN loss prevents malignant transformation in pre-B acute lymphoblastic leukemia [21]. Similarly, PTEN can enhance the stability of gain-of-function P53 mutants, thus conferring tumor-promoting functions to glioma cells [22]. During this study, an unanticipated observation emerged, wherein PTEN-L treatment resulted in distinct cellular behaviors in PTEN-deficient and PTEN-expressing tumor cells. This underscores the potentially critical role of PTEN-L in the selection of PTEN-deficient tumor cells during tumor metastasis.

Numerous studies have highlighted the link between PTEN loss and essential aspects of tumor cell behavior through the activation of downstream signaling pathways, notably the PI3K-AKT-mTOR pathway [23]. In terms of strategies for treating tumors, several PI3K and AKT inhibitors have shown promise in preclinical studies. Furthermore, PTEN reactivation in tumor cells has emerged as an attractive avenue for potential therapies. Lee et al. reported the potent antitumor effects of indole-3-carbinol, a pharmacological inhibitor of WWP1, which were achieved by reactivating PTEN [24]. Additionally, the delivery of PTEN mRNA via nanoparticle-mediated methods has restored PTEN function in PTEN-mutated prostate cancer cells and effectively inhibited tumor growth [25]. Our findings showed that PTEN-L delivery could impede AKT activation and induce cell-cycle arrest, thus suggesting a potential pro-survival effect in tumors by evading immune attack. This effect was particularly pronounced in PTEN-null tumor cells.

Emerging evidence indicates that DNA damage and mutational burden on tumor cells influence T cell infiltration and the success of immunotherapy. It has been widely accepted that genomic instability activates immune responses via production of neoantigens [26]. PTEN plays a critical role in maintaining genomic integrity [27]. Shen et al. reported that genomic and chromosomal instability was significantly increased in PTEN-null mice, and PTEN promoted double-stranded DNA break repair by regulating Rad51 expression [28]. PTEN deficiency-induced stabilization of DNA binding protein 1 facilitates TNFα transcription [29]. Similarly, we found that γ-H2AX, a sensitive molecular marker of DNA damage, was highly expressed in PTEN-null EO771 tumor cells, but it was inhibited by PTEN-L treatment. This finding implies that PTEN-L plays a protective role against PTEN in the regulation of genomic integrity and DNA repair, albeit exclusively in PTEN-null tumor cells. The nuances of this distinction warrant further in-depth investigation.

Mutations in PTEN are frequently observed in immunoresistant cancers. Previous studies have reported that PTEN deficiency facilitates the establishment of an immunosuppressive environment that is unfavorable for effective antitumor immune responses [30, 31]. However, the underlying mechanism remains unclear. PTEN loss is linked to a highly immunosuppressive tumor microenvironment as it mediates the accumulation of MDSCs, regulatory T cells, and M2 macrophages [32, 33]. Here, we observed that PTEN-L treatment induced high PD-L1 expression in PTEN-null tumor cells, which may have contributed to the escape of T cell-mediated immunosurveillance. Therefore, it is speculated that PTEN-L protects PTEN-deficient tumor cells from immune attacks during tumor progression. In addition to tumor cells, we found that PTEN-L mediates the polarization of macrophages into immunosuppressive phenotypes both in vitro and in vivo. The increased number of CD206^+^ macrophages in the lungs may provide a suitable immunosuppressive environment for circulating tumor cell colonization. In the present study, selection for PTEN-L-treated PTEN-KO EO771 cells was not observed in SCID mice. SCID mice, characterized by their lack of T and B cells, are a model of immunodeficiency. Notably, macrophages in SCID mice demonstrate enhanced expression of the inducible nitric oxide synthase pathway [34]. The observation underscores the notion that the establishment of an immunosuppressive microenvironment contingent on PTEN-L dependence contributes significantly to the selection, subsequent colonization, and growth of PTEN-deficient tumor cells in the lungs.

PTEN/PTEN-L expression is tissue-dependent. Previous studies have reported that the brain, lymph nodes, lungs, and intestines exhibit higher PTEN-L (PTENa) expression [35]. PTEN-L is a secreted protein derived from PTEN-expressing cells. Although the mechanisms underlying PTEN-L production and secretion are not fully understood, PTEN/PTEN-L levels have been reported to differ between tissues. We found that the chemotherapy drugs DDP and PTX could increase the production of PTEN/PTEN-L in the lungs, but not in the liver and spleen. Similarly, these chemotherapeutic drugs upregulated PTEN-L secretion in the supernatant of PTEN-wild-type tumor cells. Moreover, we found that these chemotherapeutic drugs upregulated PTEN expression. It has been reported that PTEN can be secreted and transported via exosomes. Whether secreted exosomal PTEN has a similar effect on PTEN-null tumor cell dormancy and growth in the lungs requires further investigation.

Tumor cell dormancy is essential for the survival of tumor cells in a new environment and initiation of metastasis. Aged lung microenvironment enables melanoma cells to enter dormancy and efficiently initiate metastatic outgrowth in the lungs by inhibiting WNT5A [36]. In this study, we found that PTEN-L treatment induced dormancy in PTEN-null tumor cells; namely, it increased cell-cycle arrest and reduced cell proliferation. Cell-cycle arrest is known to play a key role in dormancy, causing resistance to therapy while adapting to a new microenvironment, and is believed to be a factor in the formation of metastatic tumors [37]. Additionally, reduced cell proliferation can protect tumor cells from immune attacks. Our results suggest that PTEN-L-treated PTEN-null EO771 tumor cells exhibit a higher ability to survive and grow in the lungs by activating the p38 signaling pathways. Moreover, targeting PTEN-L secretion or p38 activation can inhibit the survival and growth of PTEN-null tumor cells in the lungs. Further studies are required to investigate how dormant tumor cells are reawakened in the lungs.

## Conclusions

In summary, the present study shows that PTEN-L safeguards the selection of PTEN-null tumor cells during the metastatic cascade by inducing p38-mediated tumor cell dormancy. When formulating therapeutic strategies involving PTEN-L delivery, it is imperative to consider PTEN expression and strategically focus on preventing PTEN-deficient tumor cell selection.

### Electronic supplementary material

Below is the link to the electronic supplementary material.

Supplementary Material 1

## Data Availability

The RNA sequencing data were deposited in the GEO database under the accession number GSE246600.

## Abbreviations

DDP: Cisplatin
PTX: Paclitaxel
DOX: Doxorubicin
PTEN-L: PTEN-long
SCID: Severe combined immunodeficiency
PFA: Paraformaldehyde
KO: Knockout
GSEA: Gene set enrichment analysis
