# Supplementary material for: Chemotherapy-induced PTEN-L secretion promotes the selection of PTEN-deficient tumor cells
Source: J Exp Clin Cancer Res. 2024 May 11;43:140. doi: 10.1186/s13046-024-03059-y (PMC11088029; doi:10.1186/s13046-024-03059-y)
Supplement: Supplementary file 1 — Supplementary Material 1 [file 13046_2024_3059_MOESM1_ESM.docx]

**Supplementary table 1：The antibodies employed in the flow cytometry experiments are outlined below.**

| **Antibody Name** | **Dilution** | **Catalog** | **Supplier** |
| --- | --- | --- | --- |
| CD274 (PD-L1, B7-H1)-PE | 1:100 | 12-5982-82 | Invitrogen  (Carlsbad, CA, USA) |
| CD274 (PD-L1, B7-H1)-eFluor™ 450 | 1:100 | 48-5983-42 | Invitrogen  (Carlsbad, CA, USA) |
| PerCP anti-mouse CD8a Antibody | 1:100 | 100732 | Biolegend  (San Diego,CA,USA) |
| Brilliant Violet 510™ anti-mouse CD4 Antibody | 1:100 | 100553 | Biolegend  (San Diego,CA,USA) |
| APC anti-mouse/human CD45R/B220 Antibody | 1:100 | 103212 | Biolegend  (San Diego,CA,USA) |
| FITC anti-mouse NK-1.1 Antibody | 1:100 | 108706 | Biolegend  (San Diego,CA,USA) |
| Alexa Fluor® 700 anti-mouse CD45 Antibody | 1:100 | 103128 | Biolegend  (San Diego,CA,USA) |
| PE/Cyanine5 anti-mouse I-A/I-E Antibody | 1:100 | 107612 | Biolegend  (San Diego,CA,USA) |
| PE/Cyanine7 anti-mouse F4/80 Antibody | 1:100 | 123114 | Biolegend  (San Diego,CA,USA) |
| APC/Cyanine7 anti-mouse/human CD11b Antibody | 1:100 | 101226 | Biolegend  (San Diego,CA,USA) |
| Brilliant Violet 510™ anti-mouse Ly-6G/Ly-6C (Gr-1) Antibody | 1:100 | 108437 | Biolegend  (San Diego,CA,USA) |
| PE anti-mouse CD206 (MMR) Antibody | 1:100 | 141706 | Biolegend  (San Diego,CA,USA) |
| PerCP/Cyanine5.5 anti-mouse CD206 (MMR) Antibody | 1:100 | 141716 | Biolegend  (San Diego,CA,USA) |
| APC anti-mouse/human CD11b Antibody | 1:100 | 101212 | Biolegend  (San Diego,California,USA) |
| PE anti-mouse CD163 Antibody | 1:100 | 156704 | Biolegend  (San Diego,CA,USA) |
| PE/Cyanine7 anti-mouse I-A/I-E Antibody | 1:100 | 107629 | Biolegend  (San Diego,CA,USA) |
| APC/Cyanine7 anti-mouse F4/80 Antibody | 1:100 | 123117 | Biolegend  (San Diego,CA,USA) |
| Brilliant Violet 605™ anti-mouse F4/80 Antibody | 1:100 | 123133 | Biolegend  (San Diego,CA,USA) |

**Supplementary table 2：The antibodies used in the Western blot experiments are as follows.**

| **Antibody Name** | **Dilution** | **Catalog** | **Supplier** |
| --- | --- | --- | --- |
| PTEN | 1:1000 | #9188S | Cell Signaling Technology |
| PCNA | 1:1000 | #13110S | Cell Signaling Technology |
| Na,K-ATPase | 1:1000 | #3010S | Cell Signaling Technology |
| AKT | 1:1000 | #9272S | Cell Signaling Technology |
| Phospho-Akt | 1:1000 | #4060S | Cell Signaling Technology |
| Phospho-p38MAPK | 1:1000 | #4511S | Cell Signaling Technology |
| p38MAPK | 1:1000 | #8690S | Cell Signaling Technology |
| Phospho-HistoneH2A.X | 1:500 | #80312S | Cell Signaling Technology |
| PD-L1/CD274 | 1:1000 | #A19135 | ABclonal |
| CDKN1B/p27KIP1 | 1:1000 | #A16633 | ABclonal |
| Cyclin A2 | 1:1000 | #A19036 | ABclonal |
| Cyclin B1 | 1:1000 | #A19037 | ABclonal |
| Cyclin D1 | 1:1000 | #A19038 | ABclonal |
| P16 | 1:500 | #10883-1-AP | Proteintech |
| P21 | 1:500 | sc-6246 | Santa Cruz |
| Cyclin E1 | 1:1000 | #A14225 | ABclonal |
| β-Actin | 1:8000 | #AC006 | ABclonal |
| GAPDH | 1:3000 | #AC001 | ABclonal |

**Supplementary table 3：The primer sequences employed in the RT-qPCR are as follows.**

| **Gene** | **Forward Primer** | **Reverse Primer** |
| --- | --- | --- |
| *Ccna2* | TTGTAGGCACGGCTGCTATGCT | GGTGCTCCATTCTCAGAACCTG |
| *Ccnb1* | AGAGGTGGAACTTGCTGAGCCT | GCACATCCAGATGTTTCCATCGG |
| *Ccnd1* | GCAGAAGGAGATTGTGCCATCC | AGGAAGCGGTCCAGGTAGTTCA |
| *Ccne1* | AAGCCCTCTGACCATTGTGTCC | CTAAGCAGCCAACATCCAGGAC |
| *P27* | AGATACGAGTGGCAGGAGGT | ATGCCGGTCCTCAGAGTTTG |
| *Il6* | TACCACTTCACAAGTCGGAGGC | CTGCAAGTGCATCATCGTTGTTC |
| *Il0* | CCCAAGTAACCCTTAAAGTCCTGC | ATGCTGCCTGCTCTTACTGACTG |
| *Mrc1* | GTTCACCTGGAGTGATGGTTCTC | AGGACATGCCAGGGTCACCTTT |
| *Tnfa* | GGTGCCTATGTCTCAGCCTCTT | GCCATAGAACTGATGAGAGGGAG |
| *Tgfb1* | AGCTGCTTATCCCAGATTCAGCCA | TATCGAGGCCAGCTTGTTTGAGGA |
| *Inos2* | GAGACAGGGAAGTCTGAAGCAC | CCAGCAGTAGTTGCTCCTCTTC |
| *Cd274* | TGCGGACTACAAGCGAATCACG | CTCAGCTTCTGGATAACCCTCG |
| *Actin* | GGTCCACACCCGCCACCAG | CACATGCCGGAGCCGTTGTC |


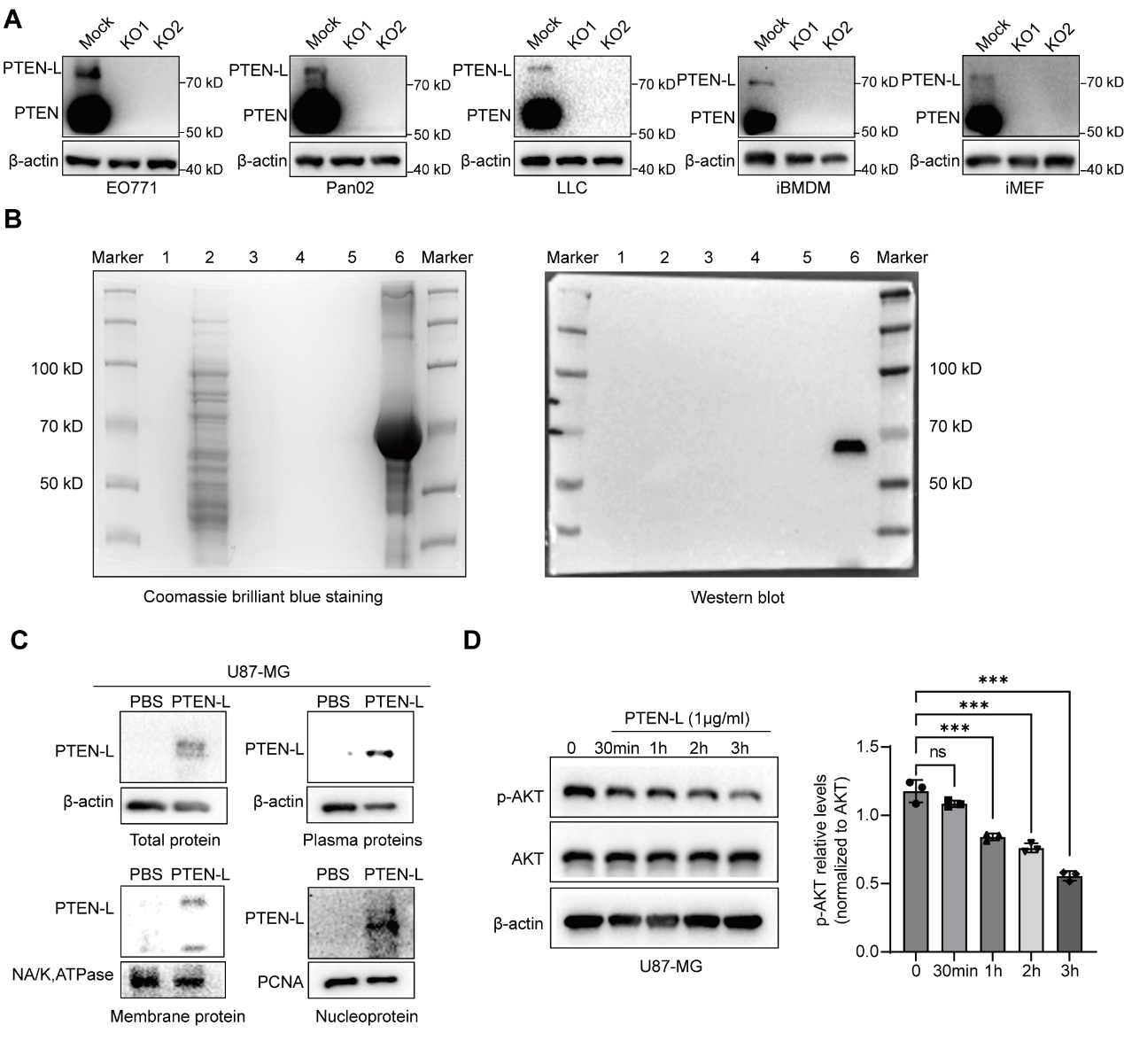


**Supplementary Figure 1. PTEN-L purification and identification.**

(A) Western blot analysis was conducted to assess PTEN expression in Mock, KO 1, and KO 2 cell clones during the establishment of PTEN-knockout cell lines (EO771, Pan02, LLC, iBMDM, and iMEF) utilizing the CRISPR-cas9 system. The knockout effects in two colonies were evaluated, with representative images displayed. β-actin served as the loading control. (B-C) The recombinant PTEN-L-6-His-tag protein was purified using immobilized NiSepharose affinity chromatography. The purity of PTEN-L was confirmed through Coomassie brilliant blue staining and western blotting. Line 1 represents total bacterial lysis; lines 2 and 3 correspond to insoluble and soluble protein extracts, respectively; lines 4-6 depict elution fractions with 20 mM, 30 mM, and 250 mM imidazole. (D) U87-MG cells were treated with purified PTEN-L (1 μg/ml), followed by subcellular fractionation. Western blotting verified the subcellular localization of PTEN-L. (E) U87-MG cells treated with PTEN-L (1 μg/ml) were analyzed for phosphorylated and total AKT via western blot. Representative images and quantifications from three experiments are presented.


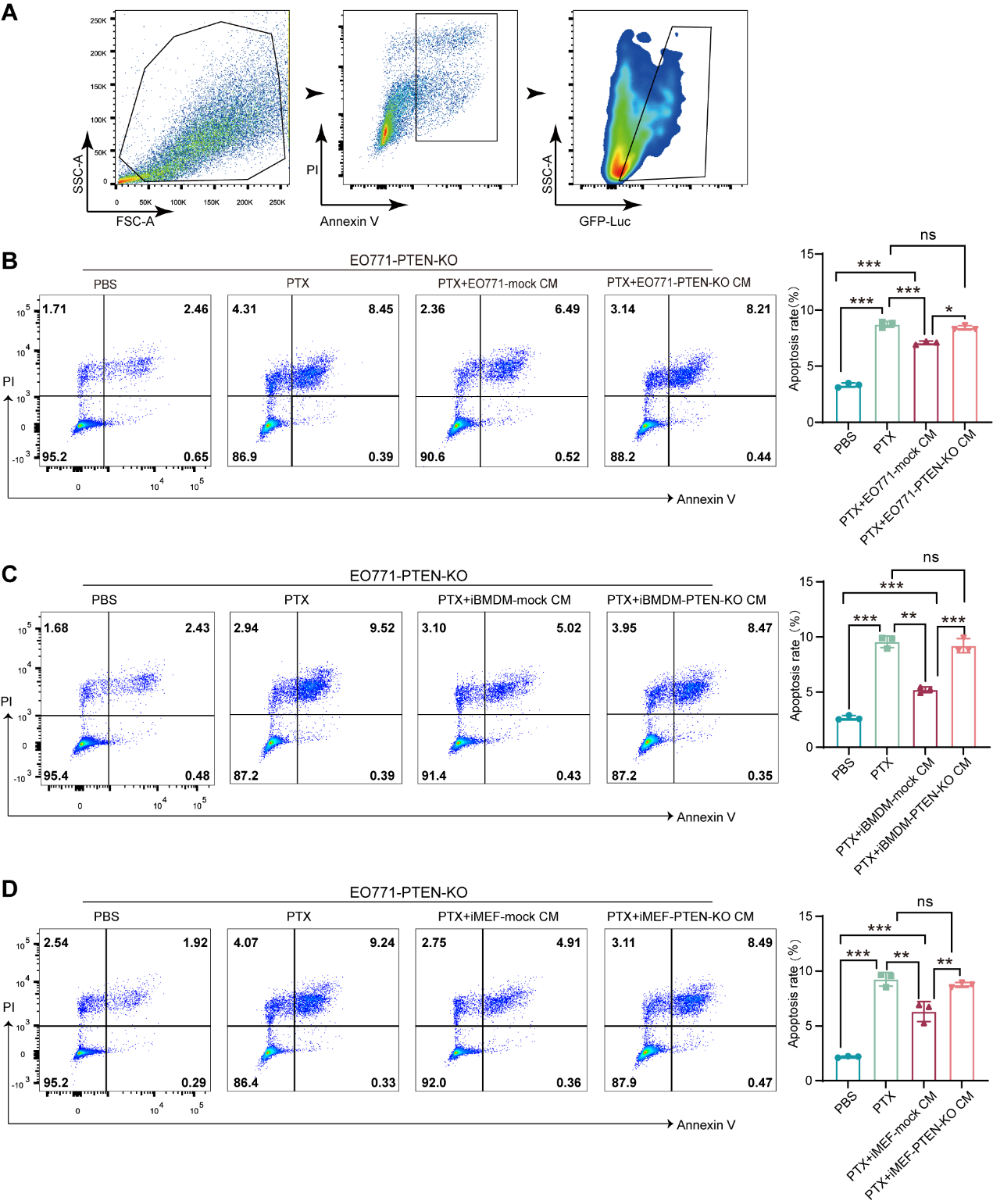
**Supplementary Figure 2. The secretion of PTEN-L protects EO771 PTEN-null tumor cells from PTX-induced apoptosis.** (A) The image displays the gating strategy used to analyze the percentage of GFP-luc cells within the annexin V-positive apoptotic cell population. (B-D) EO771-PTEN-KO cells were incubated with cell supernatant obtained from PTEN-expressing or PTEN-null cells, including EO771, iBMDM, and iMEF. Following this, the cells were treated with PTX (100nM) for 24 hours. Apoptotic cells were then quantified using flow cytometry. Representative images and bar plots summarizing the results from three independent experiments are presented (n=3, one-way ANOVA). *p < 0.05, **p < 0.01, ***p < 0.001.


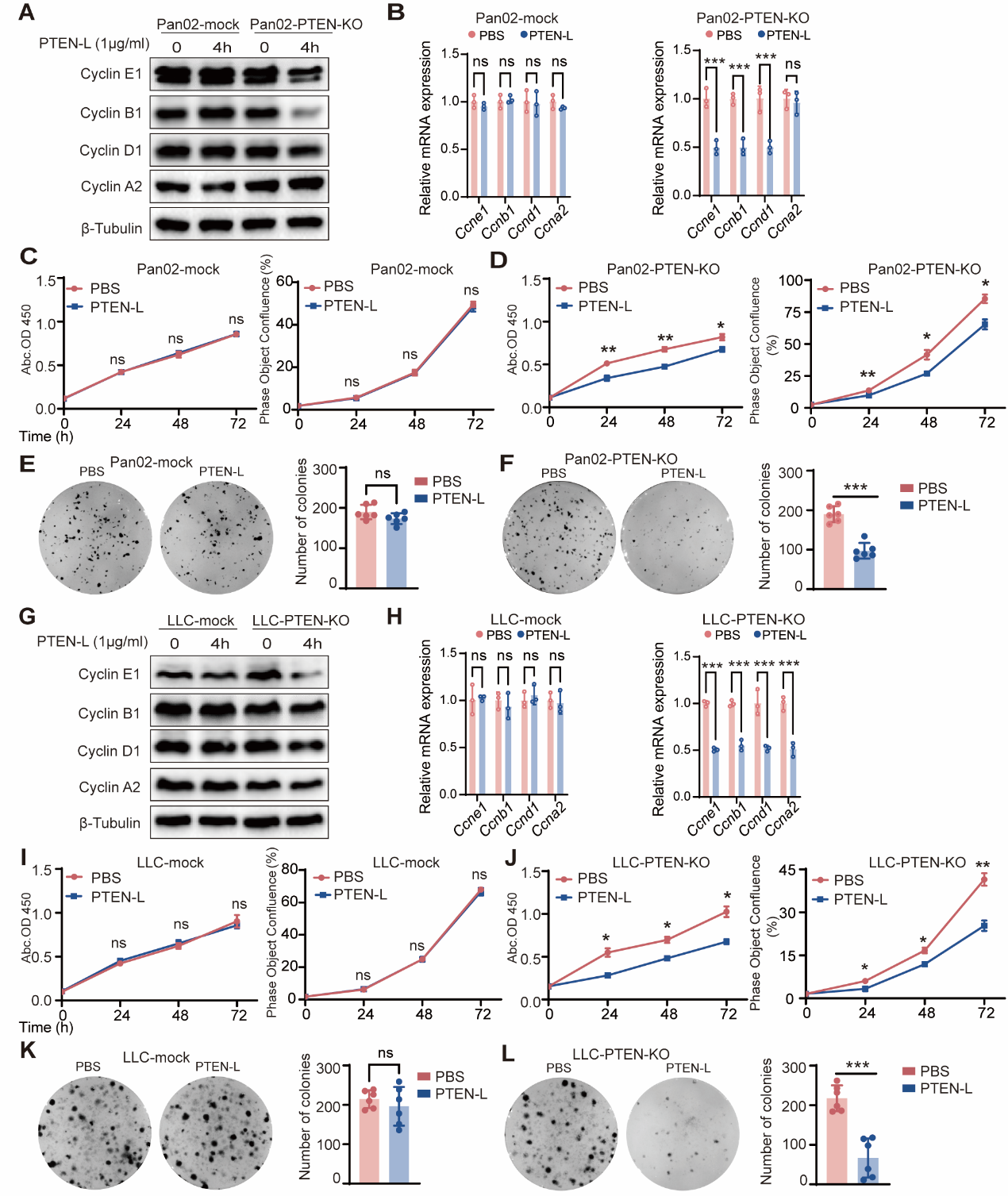


**Supplementary Figure 3. PTEN-L inhibits PTEN-null tumor cells proliferation.** A-L: LLC-mock/PTEN-KO and Pan02-mock/PTEN-KO cells underwent treatment with PTEN-L (1μg/ml) at specified time points, with PBS serving as the control. (A-B, G-H) Cyclin levels were analyzed at the protein (left) and mRNA (right) levels through western blot and PCR analysis. (C-D, I-J) The effect of PTEN-L on the proliferation of various tumor cells was evaluated using CCK8 assays (left) and cell fusion assays (right) (n = 3, two-way ANOVA). Significance levels are denoted as *p < 0.05, **p < 0.01. (E-F, K-L) Clonogenic assays were performed to investigate the influence of PTEN-L on cell proliferation (n = 6), Welch’s t-test, ***p < 0.001, ns: not significant.


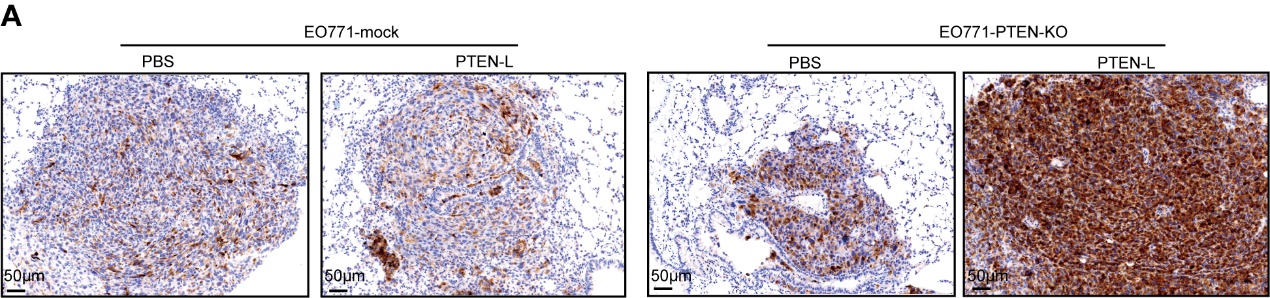


**Supplementary Figure 4. PTEN-L upregulate PD-L1 expression in lung metastasis node of PTEN-L treated EO771-PTEN-KO cells. (**A) Lung metastatic nodules from EO771-mock and EO771-PTEN-KO cells were collected after PTEN-L treatment, and immunohistochemical staining, along with imaging, revealed the expression of PD-L1 at the tumor sites.


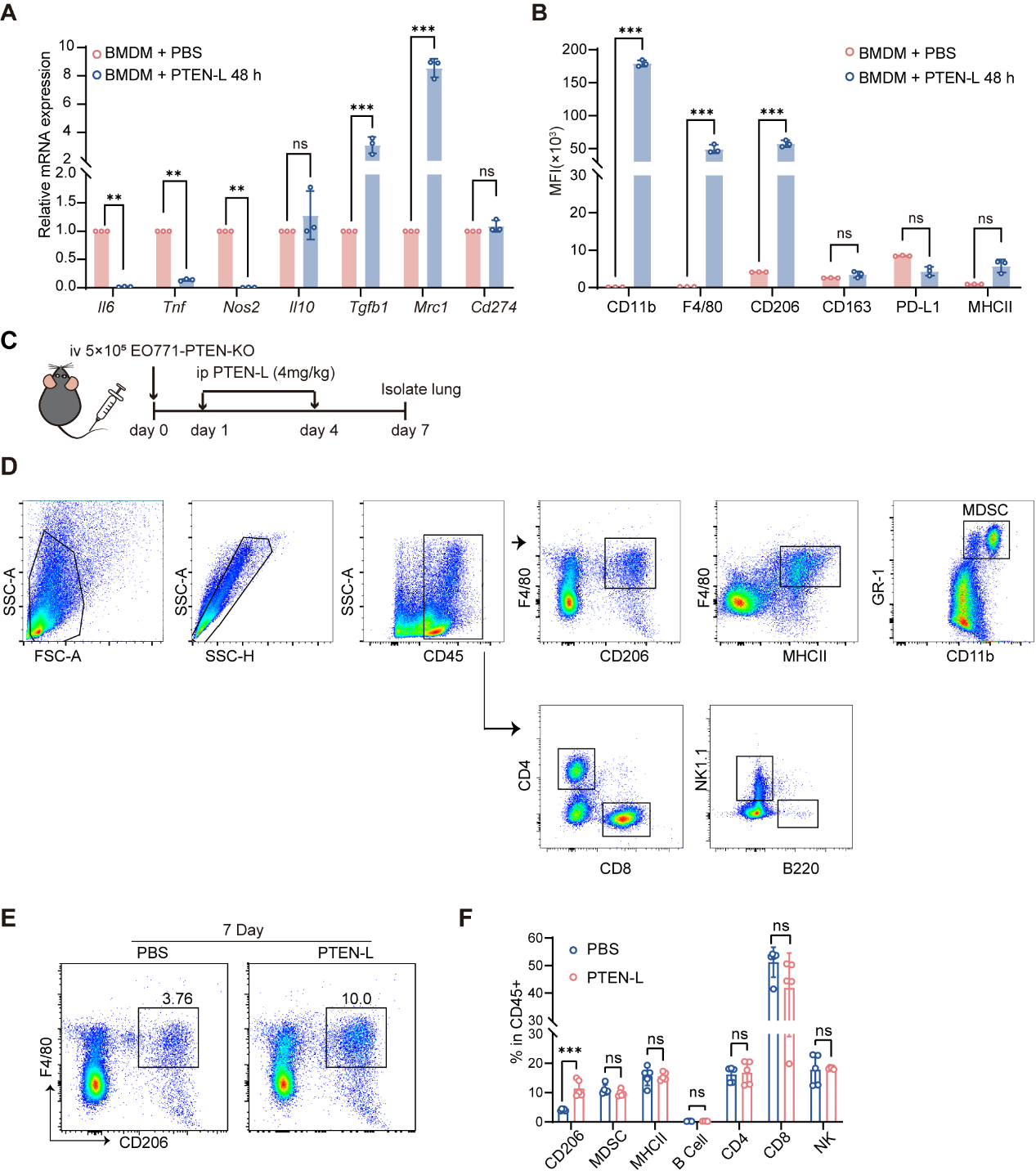


**Supplementary Figure 5. PTEN-L induces macrophages to polarize towards an immunosuppressive phenotype.** (A, B) BMDMs were incubated with PTEN-L (1 μg/ml) for 48 hours. (A) RT-PCR was utilized to detect the expression of phenotype-related genes. (B) Flow cytometry measured the mean fluorescence intensity (MFI) of macrophage markers (n=3). C-F: A total of 5 *10^5^ EO771-PTEN-KO cells were injected into the tail vein of C57BL/6J mice, followed by PTEN-L treatment (4 mg/kg) every 3 d. (C) The experimental design is outlined. (D) The gating strategy for identifying immune cells in the lungs is depicted. (E) Representative images display CD206^+^ macrophages in two groups. (F) Bar plots summarize the percentage of immune cells in both groups (n = 5). A, B and F, two-way ANOVA, **p<0.01; ***p<0.001.


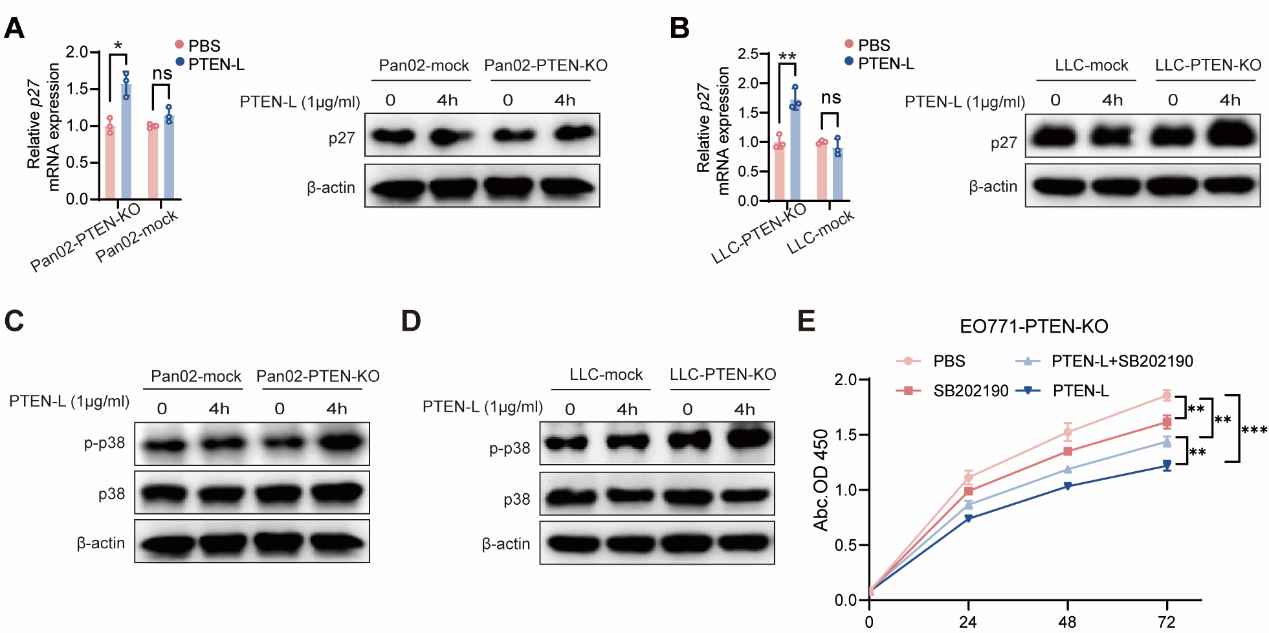


**Supplementary Figure 6. PTEN-L activates the p38 signaling pathway and upregulates the expression of p27.** Pan02-mock/PTEN-KO and LLC-mock/PTEN-KO tumor cells underwent PTEN-L treatment (1 μg/ml) at various time points. (A-B) The relative mRNA levels of p27 (left) and protein levels of p27 (right) were quantified using RT-PCR and western blot, respectively. (C-D) The expression of phosphorylated p38 (p-p38) and total p38 in tumor cells was assessed through immunoblotting. Representative images are provided. (E) EO771-PTEN-KO cells were treated with PTEN-L (1 μg/ml), SB202190 (100nM), or a combination of both, and cell proliferation was evaluated using CCK8 assays (n=3) with two-way ANOVA. The results showed significant differences where *p < 0.01, **p < 0.001.
